# A Gender-Based Analysis of High School Athletes Using Computerized Electrocardiogram Measurements

**DOI:** 10.1371/journal.pone.0053365

**Published:** 2013-01-02

**Authors:** Nikhil Kumar, Divya Saini, Victor Froelicher

**Affiliations:** 1 Henry M. Gunn High School, Palo Alto, California, United States of America; 2 Palo Alto Veterans Affairs Medical Center, Palo Alto, California, United States of America; 3 Stanford University, Palo Alto, California, United States of America; University of Virginia Health System, United States of America

## Abstract

**Background:**

The addition of the ECG to the preparticipation examination (PPE) of high school athletes has been a topic for debate. Defining the difference between the high school male and female ECG is crucial to help initiate its implementation in the High School PPE. Establishing the different parameters set for the male and female ECG would help to reduce false positives. We examined the effect of gender on the high school athlete ECG by obtaining and analyzing ECG measurements of high school athletes from Henry M. Gunn High School.

**Methods:**

In 2011 and 2012, computerized Electrocardiograms were recorded and analyzed on 181 athletes (52.5% male; mean age 16.1±1.1 years) who participated in 17 different sports. ECG statistics included intervals and durations in all 3 axes (X, Y, Z) to calculate 12 lead voltage sums, QRS Amplitude, QT interval, QRS Duration, and the sum of the R wave in V5 and the S Wave in V2 (RS Sum).

**Results:**

By computer analysis, we demonstrated that male athletes had significantly greater QRS duration, Q-wave duration, and T wave amplitude. (P<0.05). By contrast, female athletes had a significantly greater QTc interval. (P<0.05).

**Conclusion:**

The differences in ECG measurements in high school athletes are strongly associated with gender. However, body size does not correlate with the aforementioned ECG measurements. Our tables of the gender-specific parameters can help facilitate the development of a more large scale and in-depth ECG analysis for screening high school athletes in the future.

## Introduction

Sudden cardiac deaths have tragically occurred in high school athletes, who are dying suddenly while playing sports [1]. These students are usually in their mid- to late-teens and play high intensity sports, such as basketball and football. Their deaths are often attributed to heart defects that could not be detected by a routine checkup [2].

Cardiology experts agree that the preparticipation examination should include cardiovascular screening and a physical examination in the hopes of diagnosing any heart defects and diseases before the athlete takes the risk of participating in the high intensity sport [2]. The addition of a resting 12-lead electrocardiogram to the PPE, however, remains controversial. There is a debate in the medical community on the prohibitive costs (both from performing ECGs, as well as the secondary costs of additional testing), validity of results, as well as the impracticality associated with large-scale ECG testing. Despite the controversy, some countries outside the United States have implemented cardiac testing via ECG measurements at all levels of sports – from high school to professional. For example, in Italy, a screening program for competitive athletes was mandated by law, and has been implemented since 1982, thereby suggesting that it is possible to implement large scale ECG testing [3], [4].

Adding the ECG to the PPE in the United States, as well as other countries, however, brings up the question of the effect of gender on “normal” computerized measurements for the development of automated ECG interpretation for screening teens.

The question arises regarding whether the standard ECG measurements should vary for male and female athletes. This paper will investigate the relationship between gender and ECG measurements as well as the similarities and differences between these measurements on high school level athletes. The ultimate goal is to design a set of “normal” measurements for males and females, if a difference between the two exists, at the high school level. It is vital to ensure that these measurements will lead to a low false-positive rate but will still be effective in detecting heart defects before players go onto the field, thus, potentially saving many young lives.

## Methods

### Ethics Statement

The Institutional Review Board at Stanford University specifically approved this study. Written Consent was received from all athletes and their parent/next of kin before any involvement in the study could take place.

The target population was 9^th^ through 12^th^ grade high school students at Henry M. Gunn High School currently participating in high school level sports. To participate in the high school sports programs, high school athletes do not currently undergo any testing other than a simple physical exam performed by a pediatrician. [5]

To attempt to develop a sample of male and female athletes, an article was placed in the school newspaper about the heart risks that are associated with sports, as well as the significance of getting an ECG. Anyone who was interested was contacted and given a consent form. Afterwards, the athletic director was contacted, and his/her permission was obtained to meet with the athletes and perform the ECGs on the school campus after school.

Computerized ECGs were recorded and analyzed on 181 athletes (52% male; mean male age, 16.3±0.9 years; mean female age, 16.0±1.3) representing 17 sports. The 17 different sporting disciplines included: softball/baseball, golf, wrestling, martial arts, basketball, crew/rowing, cross-country, lacrosse/soccer, gymnastics, swimming/diving, tennis, track and field, volleyball, water polo, badminton, football linemen and football other.

Between 2011 and 2012, ECGs were recorded using Galix ECG machines by trained individuals, on consented athletes, and digital recordings were analyzed using Cardea Associates, Inc software and entered into a database. The ECGs were read and interpreted by an experienced cardiologist (VFF).

This investigation was designed as a study of ECG findings in high school athletes. Athletes judged to have significant abnormalities by the senior investigator were recommended to undergo further testing, namely the echocardiogram.

ECG measurements included all intervals and durations taken from the 12 leads. Values that were considered improbable were corrected after visual examination. Intervals/durations were presented in milliseconds (ms), while amplitudes are presented in microvolts (µV). P waves, T waves, and QRS complex voltages are represented in spatial constructs. The values used for determining left ventricular dilation/hypertrophy were considered, and are described in the following algorithms [4], [6].

- RSsum;
- 12-lead voltage sum for QRS amplitude;
- 12-lead voltage sum for QRS area (QRS area for each lead was calculated as: (Q amplitude×Q duration)/2+ (R amplitude×R duration)/2+ (S amplitude×S duration)/2); QRS area for all 12 leads was summed to obtain 12-lead voltage sum for QRS area).

**Figure 1:**
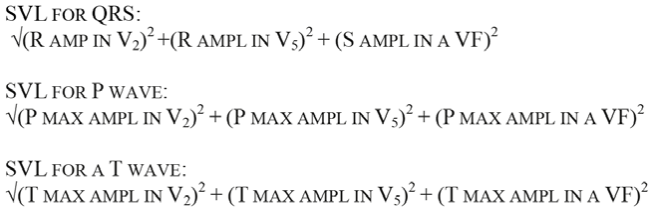
Spatial vector length (SVL) formulas for P-wave, T-wave, and QRS amplitudes. These computations work to solve for the Spatial Vector Length, which represents the maximum electrical energy emitted by the heart.

Spatial vector length (SVL) for P-wave, T-wave and QRS complex (Figure 1. Spatial vector length (SVL) calculations used for P-wave, T-wave, and QRS amplitudes). This calculation was used because it represents the maximum electrical energy generated by the heart and obviates the need to consider individual leads.

Other ECG parameters included PR interval, QRS duration, QTc interval, P axis, QRS axis, and T axis. Data were analyzed using NCSS Statistical Software (NCSS, Kayesville, UT).

Differences between the groups were compared using unpaired T-Tests. Since most ECG measurements were non-Gaussian, all data were presented as medians. Correlations were calculated between the demographics and key ECG statistics. Age was not chosen due to the small age range in the sample. Gender, height, weight, body mass index (BMI), and race were the independent variables.

A survey comprising of the AHA ECG questions was conducted before each ECG test in order to gauge the high school athlete population regarding family history or potential issues with exercise.

1. Do you experience chest pain while exercising?
2. Have you ever lost consciousness while exercising?
3. Have you ever been short of breath or had fatigue with exercise?
4. Have you ever been diagnosed with a heart murmur?
5. Has anyone in your family been diagnosed with Marfan’s, cardiomyopathy, or any other cardiac disease?
6. Has anyone in your family under the age of 50 died of unexpectedly of a cardiac disease?
7. Has anyone in your family under the age of 50 been disabled by a cardiac disease?

Results are analyzed in the next section.

## Results

The High School athlete sample rarely showed ECG abnormalities (Table 1). Normal ECGs were found in 97% of males and 97% of females. However, 57% of male athletes and 80% of female athletes had a negative response to any of the AHA ECG questions (Table 2). However, only 3% of male athletes and 0% of female athletes had 1+ positive responses and 1+ ECG abnormalities. 59% of male athletes and 71% of female athletes did not have a positive response to the questions, nor did they have abnormal ECGs. We can see that the rate of positive responses of the questions was much higher than the rate of ECG abnormalities in our high school athlete sample (Table 3).

**Table 1:** The instances of ECG abnormalities based on gender and race.

ECG Abnormality	Total (181)	Male (95)	Female (86)	Asian (60)	Caucasian(106)	Hispanic (9)	African American(6)
Right Atrial Abnormality	1 (0.6%)	1 (1.1%)	0 (0.0%)	0 (0.0%)	1 (0.9%)	0 (0.0%)	0 (0.0%)
Left Atrial Abnormality	1 (0.6%)	1 (1.1%)	0 (0.0%)	0 (0.0%)	1 (0.9%)	0 (0.0%)	0 (0.0%)
Atrial Flutter	0 (0%)	0 (0%)	0 (0.0%)	0 (0.0%)	0 (0.0%)	0 (0.0%)	0 (0.0%)
Atrial Fibrillation	0 (0%)	0 (0%)	0 (0.0%)	0 (0.0%)	0 (0.0%)	0 (0.0%)	0 (0.0%)
Long QRS duration	0 (0%)	0 (0%)	0 (0.0%)	0 (0.0%)	0 (0.0%)	0 (0.0%)	0 (0.0%)
PVCs	1 (0.6%)	0 (0%)	1 (1.2%)	0 (0.0%)	1 (0.9%)	0 (0.0%)	0 (0.0%)
PR prolongation	0 (0%)	0 (0%)	0 (0.0%)	0 (0.0%)	0 (0.0%)	0 (0.0%)	0 (0.0%)
Left Axis Deviation	1 (0.6%)	0 (0%)	1 (1.2%)	0 (0.0%)	1 (0.9%)	0 (0.0%)	0 (0.0%)
Right Axis Deviation	1 (0.6%)	0 (0%)	1 (1.2%)	0 (0.0%)	1 (0.9%)	0 (0.0%)	0 (0.0%)
Left Bundle Branch Block	0 (0%)	0 (0%)	0 (0.0%)	0 (0.0%)	0 (0.0%)	0 (0.0%)	0 (0.0%)
Right Bundle Branch Block	0 (0%)	0 (0%)	0 (0.0%)	0 (0.0%)	0 (0.0%)	0 (0.0%)	0 (0.0%)
Wolff-Parkinson-White Syndrome	0 (0%)	0 (0%)	0 (0.0%)	0 (0.0%)	0 (0.0%)	0 (0.0%)	0 (0.0%)
Right Ventricular Hypertrophy	0 (0%)	0 (0%)	0 (0.0%)	0 (0.0%)	0 (0.0%)	0 (0.0%)	0 (0.0%)
Left Ventricular Hypertrophy	0 (0%)	0 (0%)	0 (0.0%)	0 (0.0%)	0 (0.0%)	0 (0.0%)	0 (0.0%)
Brugada	0 (0%)	0 (0%)	0 (0.0%)	0 (0.0%)	0 (0.0%)	0 (0.0%)	0 (0.0%)
ST Depression	0 (0%)	0 (0%)	0 (0.0%)	0 (0.0%)	0 (0.0%)	0 (0.0%)	0 (0.0%)
Long QTc	0 (0%)	0 (0%)	0 (0.0%)	0 (0.0%)	0 (0.0%)	0 (0.0%)	0 (0.0%)
T wave Inversion in V5	1 (0.6%)	0 (0%)	1 (1.2%)	1 (1.7%)	0 (0.0%)	0 (0.0%)	0 (0.0%)
T wave Inversion in AVF	0 (0%)	0 (0%)	0 (0.0%)	0 (0.0%)	0 (0.0%)	0 (0.0%)	0 (0.0%)
T wave Inversion in V2	2 (1.2%)	2 (2.1%)	0 (0.0%)	0 (0.0%)	2 (1.9%)	0 (0.0%)	0 (0.0%)
Athletes with more than one of theabove Abnormality	1 (0.6%)	1 (1.1%)	0 (0.0%)	0 (0.0%)	1 (0.9%)	0 (0.0%)	0 (0.0%)
Normal ECG	175 (96.7%)	92 (96.8%)	83 (96.5%)	59 (98.3%)	100 (94.3%)	9 (100%)	6 (100%)

**Table 2:** Responses to the AHA 12 Points for Cardiac Risk according to race and gender.

Cardiac History/Family Cardiac History	Total (181)	Male (95)	Female (86)	Asian (60)	Caucasian (106)	Hispanic (9)	African American (6)
Experience Chest Pain During Exercise	16 (8.8%)	10 (10.5%)	6 (7.0%)	5 (8.3%)	8 (7.5%)	3 (33.3%)	0 (0%)
Lost Consciousness During Exercise	15 (8.3%)	5 (5.3%)	10 (11.6%)	7 (8.2%)	7 (6.6%)	1 (11.1%)	0 (0%)
Short of Breath or Fatigue with Exercise	20 (11.1%)	12 (6.6%)	8 (9.3%)	5 (5.85)	13 (12.3%)	2 (22.2%)	0 (0%)
Heart Murmur	4 (2.2%)	3 (1.7%)	1 (1.2%)	0 (0%)	3 (2.8%)	1 (11.1%)	0 (0%)
High Blood Pressure	1 (0.5%)	0 (0%)	1 (1.2%)	0 (0%)	1 (0.9%)	0 (0%)	0 (0%)
Family History of Heart Disease	20 (11.1%)	16 (16.8%	4 (4.7%)	7 (8.2%)	11 (10.3%)	2 (22.2%)	0 (0%)
Family History of Sudden Cardiac Death	15 (8.3%)	10 (10.5%)	5 (5.8%)	3 (3.5%)	11 (10.3%)	0 (0%)	1 (16.7%)
Family History of Being Disabled by Heart Disease	17 (9.4%)	16 (16.8%)	1 (1.2%)	3 (3.5%)	11 (10.3%)	2 (22.2%)	1 (16.7%)
**Athletes with more than** **one of the above**	30 (16.6%)	25 (26.3%)	5 (5.8%)	10 (11.6%)	16 (15.1%)	3 (33.3%)	1 (16.7%)

**Table 3:** Comparison of ECG abnormalities based on responses to AHA ECG questions.

Response to Questions/ECG abnormalities	Total (181)	Male (95)	Female (86)	Asian (60)	Caucasian (106)	Hispanic (9)	African American (6)
Athletes with 0 Positive Responses to Questions	122 (67.4%)	54 (56.8%)	68 (79.7%)	41 (68.3%)	72 (67.9%)	4 (44.4%)	5 (83.3%)
Athletes with 1 Positive Response to Questions	29 (16.0%)	16 (16.8%)	13 (15.1%)	9 (15.0%)	18 (17.0%)	2 (22.2%)	0 (0,0%)
Athletes with 2+ Positive Responses to Questions	30 (16.6%)	25 (26.3%)	5 (5.8%)	10 (11.6%)	16 (15.1%)	3 (33.3%)	1 (16.7%)
Athletes with 0 Detected ECG abnormalities	175 (96.7%)	92 (96.8%)	83 (96.5%)	59 (98.3%)	100 (94.3%)	9 (100%)	6 (100%)
Athletes with 1 Detected ECG abnormalities	5 (2.8%)	2 (2.1%)	3 (3.5%)	1 (1.67%)	4 (3.8%)	0 (0.0%)	0 (0.0%)
Athletes with 2+ Detected ECG abnormalities	1 (0.6%)	1 (1.1%)	0 (0.0%)	0 (0.0%)	1 (0.9%)	0 (0.0%)	0 (0.0%)
Athletes with 0 ECG abnormalities and 0 Positive Responses	117 (64.6%)	56 (59.0%)	61 (70.9%)	40 (66.6%)	68 (64.2%)	4 (44.4%)	5 (83.2%)
Athletes with 1+ ECG abnormalities and 1+ Positive Responses	3 (1.7%)	3 (3.2%)	0 (0.0%)	0 (0.0%)	2 (1.9%)	0 (0.0%)	1 (16.7%)

Height, weight, and BMI were greater in male compared with female athletes (Table 4). There was not much difference in the percentage of ethnicities represented in each gender. Besides for sports restricted solely to males (football) and to females (gymnastics and volleyball), percentage of male and female athletes was not significantly different across the various sport disciplines. Table 5 illustrates the breakdown of sports played.

**Table 4:** Demographic Characteristics of the High School Athletes at Henry M. Gunn High School.

Variable	Total	Male	Female	P value
	Median	Median	Median	T Test
	(n = 181)	(n = 95)	(n = 86)	
Age (y)	16.1±1.1	16.3±0.9	16.0±1.3	
Ethnicity (n) %				
Caucasian	106 (58.6%)	50 (52%)	56 (65.1%)	
Asian	60 (33.1%)	37 (39%)	23 (26.7%)	
Hispanic	9 (5%)	6 (6%)	3 (3.5%)	
African American	6 (3.3%)	2 (2%)	4 (4.7%)	
Height (m)	1.72±0.09	1.76±0.09	1.67±0.08	<0.05
Weight (kg)	58.6±10.4	68.68±12.67	57.7±9.43	>0.05
BMI	21.5±3.0	22.2±3	20.7±2.7	<0.05

**Table 5:** Major Sports Participated in by Gender.

Sport	Total (N)	Males (N)	Females (N)
Baseball	5 (2.8%)	2 (2.1%)	3 (3.5%)
Football (Linemen)	7 (3.9%)	7 (7.4%)	0 (0.0%)
Golf	2 (1.1%)	0 (0.0%)	2 (2.3%)
Wrestling	13 (7.2%)	12 (12.6%)	1 (1.2%)
Basketball	14 (7.7%)	6 (6.3%)	8 (9.3%)
Crew/Rowing	1 (0.6%)	0 (0.0%)	1 (1.2%)
Cross-Country	2 (1.1%)	0 (0.0%)	2 (2.3%)
Field Hockey/Lacrosse/Soccer	22 (18.2%)	18 (18.9%)	15 (17.4%)
Football (Other)	21 (11.6%)	21 (22.1%)	0 (0.0%)
Gymnastics	12 (6.6%)	0 (0.0%)	12 (14.0%)
Swimming/Diving	13 (7.2%)	8 (8.4%)	5 (5.8%)
Tennis/Squash	16 (8.8%)	8 (8.4%)	8 (9.3%)
Track and Field	9 (5%)	5 (5.3%)	4 (4.7%)
Volleyball	23 (12.7%)	0 (0.0%)	23 (26.7%)
Water Polo	7 (3.9%)	6 (6.3%)	1 (1.2%)
Other	3 (1.7%)	2 (2.1%)	1 (1.2%)
**Total**	181	95	86

Visual analysis by the senior cardiologist found no significant difference in the prevalence of abnormal ECGs between the races represented in our sample. Furthermore, detailed analysis of the computerized measurements failed to demonstrate any statistically significant differences between the races.

Table 6 describes the ECG statistics of the total high school athlete population, as well as gender. The measurements showed that males had significantly larger QRS durations, Q-wave durations, and T wave amplitudes, while significantly shorter QTc intervals in comparison to female athletes. Q-wave duration in leads I, aVF, and V5 were significantly greater in male compared with female athletes, Q-wave amplitude in leads I and V5, and T-wave amplitude in aVF, V2, and V5 were also significantly greater in male compared with female athletes.

**Table 6:** Electrocardiogram Data in Male and Female Athletes.

Variable	Total		Male		Female		P Value
	Median	IQR	Median	IQR	Median	IQR	
	(n = 181)		(n = 95)		(n = 86)		
**Major durations/intervals (ms)**							
PR interval	144	130–160	146	134–162	140	128	<.001
P-wave duration	96	88.5–106	102	94–112	93	83–101	<.001
QRS duration	92	87.5–100	98	92–104	88.6	83.0–93.6	<.001
QTc	426.4	405.4–441.0	417.2	396.6–429.2	436.6	423.7–450.5	<.001
**Axis (°)**							
P axis	47.2	28.75–58.9	54.8	44.5–60.3	38.65	17.025–48.225	<.001
QRS axis	80.4	57.7–103	65	30.1–91.3	92	74.425–111.475	<.001
T axis	31.2	24.35–39.2	28.2	21.4–32.9	38.7	29.875–45.3	<.001
**P-wave amplitudes (mv)**							
Greatest positive in II, aVF	81.5	45.25–112.1	101.3	65.6–125.7	69.9	35.975–93.725	<.001
**Q waves**							
Duration V_5_ (msec)	14.3	0–19.3	16.3	11–21.1	10.5	0–17.3	<.001
Duration aVF (msec)	14.5	0–20.05	15.1	0–21.5	14.3	0–18.5	<.001
Amplitude V_5_ (mv)	−51.7	−105.7 to 0	−66.6	−136.3 to −31.7	−36.05	−80.45 to 0	<.001
Amplitude aVF (mv)	−59.3	−121.7 to 0	−67.1	−123.6 to 0	−48.4	−118.85 to 0	<.001
**RS-wave amplitudes (mv)**							
R wave max V_5_ or V_6_	1747.1	1390.85–2229.45	1988.9	1558.9–2411.4	1523.1	1279.075–1977.225	<.001
S wave max V_2_ or V_3_	−1435.2	−1877.4 to 893.65	−1462.6	−2218.5 to −812.2	−1387.35	−1692 to −999.8	<.001
**T-wave amplitude (mv)**							
aVF	270.3	188.2–344.45	290.1	193.4–345.9	250.05	180.65–337.3	<.001
V_2_	285.2	172.1–484.2	312.5	144.8–575.6	270.85	187.5–440.1	<.001
V_5_	489.3	398.25–636.1	511.5	420.8–658.6	472.45	374.325–615.575	<.001

**Figure 2:**
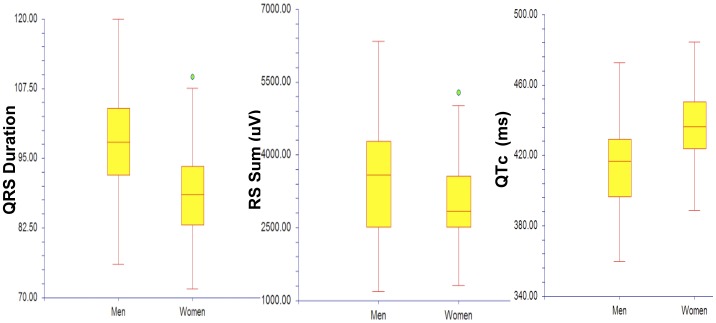
Gender differences in QRS duration, QTc, and RS Sum (S wave in V2+ R wave in V5). Measurements of the QRS Duration, QTc, and RS Sum illustrated significant differences in values between male and female high school athletes. Men have a significantly longer QTc interval, a larger RS Sum, and a shorter QRS Duration.

The voltage algorithms calculations for the P and T waves, and the QRS complex are presented in Table 7. The RS Sum, the R-wave amplitude, Q wave duration, and T wave duration is higher in males than females, while QT and QTc durations in females are higher than those of males (Figure 2).

**Table 7:** Results of the Analysis of the Summation Algorithms and SVL Calculations Representing the Maximal Electrical Energy of the Left Ventricle and Atria.

Variable	Total		Male		Female		P Value
	Median	IQR	Median	IQR	Median	IQR	
	(n = 181)		(n = 95)		(n = 86)		
**Summations**							
R wave V_5_ and	3133.1	2510.65–4009.45	3603.1	2507.7–4286.9	2856.85	2508.175–3560.1	<.001
S wave V2 (µv)							
R wave amplitude	1747.1	1390.85–2229.45	1988.9	1558.9–2411.4	1523.1	1279.075–1977.225	<.001
12 leads (µv)							
**SVL (µv)**							
R wave	2555.3	2088.65–3191.05	2822.1	2200.3–3358.9	2378.8	1957.125–3037.275	<.001
P wave	96	88.3–106	102	94–112	92.6	82.6–100.6	<.001
T wave	906	888–930	898	880–912	920	902–942	<.001

**Abbreviations:** IQR, interquartile range; SVL, spatial vector length.

In addition, the results of regression analysis of selected ECG measurements by gender with height and BMI as the independent variable are presented in Table 8. QRS duration differed significantly in men and women with regard to height (<0.001 for both).

**Table 8:** Selected ECG Measurements With Results of Regression Analysis with Height and BMI as Independent Variables for Male and Female High School Athletes.

ECG Variable	Gender	Change Per Inch (Height)	Correlation r	P Value	Change Per Unit BMI	Correlation r	P Value
QRS duration (ms)	Men	0.66	0.06	<.001	0.62	0.19	<.001
	Women	0.94	0.05	<.001	0.82	0.29	<.001
QTc (ms)	Men	−0.03	0.004	<.001	0.19	0.02	<.001
	Women	−0.58	0.09	<.001	−0.44	0.05	<.001
SVL (mv)	Men	12.6	0.05	<.001	−11.2	0.04	<.001
	Women	24.7	0.12	<.001	−28.5	0.11	<.001

Men demonstrated a 0.26 ms increase in the QRS wave per centimeter of height, while women demonstrated a 0.37 ms increase in the QRS wave per centimeter of height.

In a multiple regression analysis using gender and anthropometric variables (height, weight, BMI), gender was the strongest independent predictor of all the ECG variables, except for QRS Duration (Height was the strongest predictor, r2 = 0.21) and Spatial Vector Length P wave (BMI was the strongest predictor, r2 = 0.18). The variances explained in each model ranged from 1% to 21%. Gender was the most significant independent predictor of all major ECG intervals (QT, QTc). Height was also a predictor of QRS duration, explaining 21% of the variation in the parameter. Table 9 shows the correlation coefficients of the relationship between the variables assessed with the ECG.

**Table 9:** Correlation Matrix of Body Characteristics and Measurements of Major Durations and Maximal Electrical Energy of Ventricle and Atria.

	Height	Weight	BMI	QRS Duration	QTc	QRS SVL	Sum R Wave Amplitude	QRS Area Sum	Sum R Wave V_5_ and S Wave V_2_	P-Wave SVL	T-Wave SVL
**Total Population**											
Height	1	0.73	0.22	0.46	−0.22	0.26	0.25	0.2	0.15	0.058	0.43
Weight	0.73	1	0.82	0.48	−0.19	0.21	0.2	0.12	0.11	0.07	0.03
BMI	−0.22	0.82	1	0.32	−0.11	0.097	0.82	0.09	0.035	0.06	0
QRS duration	0.46	0.48	0.32	1	−0.22	0.17	0.1	0.19	0.11	0.025	0.005
QTc	0.22	−0.19	−0.11	−0.22	1	−0.16	−0.15	−0.13	−0.16	0.022	0.036
QRS SVL	0.26	0.21	0.097	0.17	−0.16	1	0.96	0.75	0.77	0.3	−0.05
QRS area sum	0.2	0.12	0.09	0.19	−0.13	0.75	0.81	1	0.58	0.13	0.35
Sum R wave V_5_ and Swave V_2_	0.15	0.11	0.035	0.11	−0.16	0.77	0.74	0.58	1	0.33	−0.055
P-wave SVL	0.058	0.07	0.06	0.025	0.022	0.3	0.3	0.13	0.33	1	−0.28
T-wave SVL	0.043	0.03	0	0.005	0.036	0.05	−0.015	0.35	−0.055	−0.28	1
**Males**											
Height	1	0.74	0.32	0.25	−0.25	0.08	0.1	0.18	0.054	−0.19	0.31
Weight	0.74	1	0.84	0.26	0.025	0.29	0.38	0.058	−0.01	−0.12	0.25
BMI	0.32	0.84	1	0.2	0.023	−0.029	0.03	0.14	−0.045	0	0.08
QRS duration	0.25	0.26	0.2	1	0.003	−0.021	−0.074	0.16	−0.084	0.134	0.22
QTc	−0.025	0.025	0.023	0.003	1	0.047	0.036	−0.11	0.054	0.018	−0.11
QRS SVL	0.08	0.29	−0.029	−0.021	0.047	1	0.95	0.86	0.76	0.2	0.36
QRS area sum	0.18	0.058	0.14	0.16	−0.11	0.86	0.76	1	0.78	0.14	0.2
Sum R wave V_5_ and Swave V_2_	0.054	−0.01	−0.045	−0.084	0.054	0.76	0.76	0.78	1	0.3	−0.035
P-wave SVL	−0.19	−0.12	0	0.134	0.018	0.3	0.21	0.14	0.3	1	0.1979
T-wave SVL	0.31	0.25	0.08	0.22	−0.11	0.066	0.08	0.2	−0.035	0.1979	1
**Females**											
Height	1	0.63	0.006	0.4	0.088	0.11	0.11	−0.02	0.1	0.05	0.24
Weight	0.63	1	0.77	0.48	0.09	0.087	0.088	0.03	0.014	−0.02	0.26
BMI	0.06	0.77	1	0.29	−0.055	0.034	0.031	0.03	−0.048	−0.052	0.14
QRS duration	0.4	0.48	0.29	1	−0.075	−0.08	−0.09	0.06	−0.048	−0.21	0.29
QTc	0.088	0.09	−0.055	−0.075	1	−0.076	−0.07	0.01	0.1	0.03	0.12
QRS SVL	0.11	0.087	0.034	−0.08	−0.076	1	0.975	0.74	0.75	0.2	0.17
QRS area sum	−0.02	0.03	0.03	0.06	0.01	0.74	0.85	1	0.53	0.1	0.22
Sum R wave V_5_ and Swave V_2_	0.1	0.014	−0.048	−0.048	0.1	0.75	0.75	0.53	1	0.3	0.13
P-wave SVL	0.05	−0.02	−0.052	−0.21	0.03	0.2	0.24	0.1	0.3	1	−0.16
T-wave SVL	0.24	0.26	0.14	0.29	0.12	0.17	0.21	0.22	0.13	−0.16	1

**Abbreviations:** BMI, Body Mass Index; SVL, spatial vector length.

## Discussion

This study was intended to provide a set of high school athlete data to validate the implementation of the ECG in the high school athletic pre-participation exam. To our knowledge, this is the first large-scale ECG study conducted on high school athletes, with intent to compare male and female athletes and determine the effect of sport on the hearts of these athletes. In the past, ECG studies have been only performed on college athletes.

The present study has shown gender differences of high school athletes, as well as novel findings in the high school ECG. This study showed that the predominant gender differences in high school athletes included a significantly longer QRS Duration, Q wave duration, ST Interval, and Q wave amplitude in male athletes, and a longer QTc interval in female athletes. Males also had significantly higher Q wave and T wave amplitudes. Key measurements that indicated maximal electrical activity of the heart were all greater in male high school athletes. Male athletes exhibited significantly greater R Wave Amplitude, QRS Area, and the sum of the R wave in lead V5 and S wave in lead V2.

Although ECG measurements are correlated with gender, we attempted to find if the ECG measurements were correlated with body dimensions, as males are significantly taller, heavier, and have a greater BMI. However, there is a poor correlation between body dimensions and ECG statistics. For example, height explains only 5.6% of the variation of the QRS duration in male athletes, and 15.8% of the variation of the QRS Duration in female athletes. These small differences in QRS duration between male and female athletes attribute to the small height differences.

The ECG was accompanied with the AHA questions. The subject of the questions varied from family history to heart-related issues in the patient. Sport did not seem to have an effect on the responses to these questions. 33% of the sample had at least one positive response to any of the questions. 43% of male athletes and 21% of female athletes had more than one positive response to any of the AHA questions.

ECG abnormalities were assessed for the high school athlete sample. Sport did not have an effect on the presence or absence of these abnormalities. 3% of the sample had at least one ECG abnormality. 3% of males and 4% of females had an ECG abnormality.

One of the main concerns with the ECG is the rate of false positive, and we see that the responses to the questions result in more false positives than the ECG does [7]. We have illustrated that the frequency of positive responses to the AHA questions is much higher than the frequency of ECG abnormalities observed. We can conclude that false positives should not be an issue in the mandating of the high school ECG, since the qualitative measurements (the questions) have produced more false positives than the ECG did.

### Study Limitations

There are many factors that could have affected our sample, therefore limiting our findings. One such factor could be the fact that a significant number of volleyball players contributed to the female data, while a significant number of football players contributed to the male data. However, despite the difference in sport, the purpose of the study was not to compare measurements between male and female high school athletes, rather than between sports groups. Additionally, our analysis is dependent on one commercial computerized program, and different measurements could be possible with another program. The athletes were, however, equally distributed by gender (48% female). Finally, race was not equally sampled (58.5% Caucasian, 33.14% Asian, 5.0% Hispanic, 3.3% Afro-American), and therefore, we could not perform tests of significance to compare the differences in values between the races, due to the lack of consistency in the distribution of race.

### Conclusion

There are significant gender differences in the ECG between male and female high school athletes. This included a longer QRS duration, PR interval, ST interval, and Q wave duration in male athletes, and a longer QTc interval in female athletes. In addition, there were greater T wave and P wave amplitudes in males. ECG indicators of left ventricular chamber size, including SVL, R-wave amplitude, QRS area, and the sum of the R wave in V5 and S wave in V2 were significantly greater in male athletes. Regression analysis of ECG measurements with height, weight, and BMI as independent variables and ECG statistics as dependent variables showed that the differences in the ECG were found due to gender, and not due to the discrepancies in physiological makeup. This shows that gender-specific criteria for abnormal ECG findings are necessary to facilitate a more effective approach to the ECG screening in young athletes. This study should serve as a comprehensive benchmark for the high school athlete’s ECG. The ECG should be a part of the high school athletic PPE, since we have demonstrated the validity, as well as the plausibility of performing the ECG alongside the high school athlete PPE. If the ECG were mandated in the high school PPE, many athletic sudden cardiac deaths could be prevented.
